# Hepatoprotective Effect of Kaempferol—A Review

**DOI:** 10.3390/molecules30091913

**Published:** 2025-04-25

**Authors:** Przemysław Niziński, Anna Krajewska, Tomasz Oniszczuk, Beata Polak, Anna Oniszczuk

**Affiliations:** 1 Department of Pharmacology, Medical University of Lublin, Radziwiłłowska 11, 20-080 Lublin, Poland; przemyslawnizinski@umlub.pl; 2 Department of Comprehensive Paediatric and Adult Dentistry, Medical University of Lublin, Chodżki 6, 20-093 Lublin, Poland; anna.krajewska1@umlub.pl; 3 Department of Thermal Technology and Food Process Engineering, University of Life Sciences in Lublin, Głęboka 31, 20-612 Lublin, Poland; tomasz.oniszczuk@up.lublin.pl; 4 Department of Physical Chemistry, Medical University of Lublin, Chodżki 4a, 20-093 Lublin, Poland; beata.polak@umlub.pl; 5 Department of Inorganic Chemistry, Medical University of Lublin, Chodźki 4a, 20-093 Lublin, Poland

**Keywords:** kaempferol, antioxidants, flavonoids, liver, MASLD, fibrosis, LPS

## Abstract

Liver diseases, including chronic inflammation and related metabolic dysfunction-associated steatotic liver disease (MASLD), fibrosis and cirrhosis remain a growing global health burden. Currently, available pharmacotherapy for liver dysfunction has limited efficacy. Kaempferol, a naturally occurring flavonoid, has demonstrated significant hepatoprotective effects in preclinical models. This substance activates the SIRT1/AMPK signalling pathway, improves mitochondrial function, inhibits proinflammatory cytokine production via TLR4/NF-κB suppression and attenuates hepatic stellate cell activation by modulating the TGF-β/Smad pathway. In addition, kaempferol regulates the composition of the gut microbiota, thus improving bile acid metabolism and alleviating steatosis and fibrosis. This review presents an integrated analysis of recent in vitro and in vivo studies on the mode of action and utility of kaempferol in liver disease and hepatoprotection.

## 1. Introduction

The liver is an internal organ that accounts for approximately 2% of adult body weight. The liver plays a crucial role in many physiological processes, including nutrient metabolism, detoxification, carbohydrate, protein and lipid homeostasis, and regulation of the immune system [1,2]. Maintaining proper liver function is one of the most important issues in human health. To date, approximately 4% of deaths worldwide are caused by liver disease, accounting for approximately two million cases [3]. The most serious liver diseases are cirrhosis and its progression to liver cancer, which are characterized by high mortality. Heavy alcohol consumption, western dietary habits, viral infections and metabolic disorders are considered to be the main causes of liver dysfunction, which manifests as hepatic steatosis, fibrosis and ultimately cirrhosis and hepatocellular carcinoma (HCC) [4]. In addition to alcohol consumption, which is the main cause of cirrhosis, hepatic steatosis can also develop in people who do not drink at all. Metabolic-associated steatotic liver disease (MASLD), formerly known as non-alcoholic fatty liver disease (NAFLD), is also a very common burden, with an estimated global adult prevalence of 30%. It is also estimated that the prevalence of MASLD will continue to increase, particularly in Western countries [5]. MASLD is considered to be the first step in the development of steatohepatitis (approximately one-third of all MASLD cases will progress to metabolic-associated steatohepatitis, MASH) and ultimately to cirrhosis [6]. By 2024, there will be no targeted drugs for NAFLD. However, Resmetirom, the only drug currently approved for MASH, only alleviates the fibrotic scars in the course of MASLD/MASH without addressing the underlying causes [7,8]. On the other hand, many secondary plant metabolites, including polyphenols, are known to have pharmacological activity and may be useful in various diseases. An undoubted advantage of natural products is their abundance, relatively low cost of extraction from plant material and, in most cases, mild side effects, which make them interesting candidates for the alleviation and prevention of many diseases [9]. Polyphenols such as resveratrol, chlorogenic acid (CGA), quercetin or kaempferol are considered promising molecules in hepatoprotection, mainly due to their antioxidant, anti-inflammatory, cardioprotective and antihypertensive properties [10,11,12,13]. Kaempferol, a secondary plant metabolite belonging to the flavonoids, a subclass of polyphenols, has been widely used in traditional medicine as an antioxidant, anti-inflammatory, anti-obesity, anti-ageing, chemopreventive and anti-tumour agent, as well as an antidiabetic and antihypertensive compound [14]. Kaempferol’s potential for treating central nervous system disorders is shown in promising studies [15]. Its presence in common edible and medicinal plants such as the tea plant and green and leafy vegetables makes it readily available and affordable for use in common diseases [16]. This review summarises the current state of knowledge on the potential use of kaempferol in hepatoprotection, the molecular mechanisms underlying the efficacy of kaempferol in liver diseases, and future directions for the use of kaempferol, in particular, novel dosage forms and improved bioavailability.

## 2. General Overview of Liver Disease Pathophysiology

Liver disease encompasses a spectrum of conditions that share common pathophysiological mechanisms such as oxidative stress, chronic inflammation, lipid accumulation, hepatocyte death and fibrosis. These complex and interrelated processes can lead to progressive impairment of liver structure and function, which can manifest as cirrhosis and hepatocellular carcinoma (HCC) [17]. Due to the increasing prevalence of metabolic disorders such as diabetes mellitus, dyslipidaemia, or obesity, metabolic-associated steatotic liver disease (MASLD), formerly known as non-alcoholic fatty liver disease (NAFLD), is becoming a major concern [18]. MASLD, a term that encompasses a spectrum of symptoms, is caused by lipid accumulation in hepatocytes. The term NAFLD has been utilised to describe the histological spectrum from steatosis to steatohepatitis. Nevertheless, the utilisation of the term NAFLD is not without its limitations, as it is predicated on exclusionary confounding terminology and employs language that has the potential to engender stigma. In 2023, a consensus was reached that the acronym should be revised to MASLD, which signifies ‘metabolic dysfunction-associated steatotic liver disease’ [18]. It is primarily caused by alterations in carbohydrate or lipid metabolism, which are often associated with other metabolic diseases, so MASLD is often considered to be the hepatic manifestation of the metabolic syndrome [19]. Other possible mechanisms for the development of MASLD have been proposed, including an imbalance in the gut microbiota, sleep disturbances and thyroid hormone alterations [20,21,22]. MASLD is the first step, albeit fully reversible in the early stages, in the progression to metabolic dysfunction-associated steatohepatitis (MASH) with varying degrees of fibrosis. Further progression includes cirrhosis and HCC [23,24,25]. In general, fibrosis can be either metabolic or iatrogenic and is the result of untreated chronic hepatic inflammatory processes in which the liver constantly attempts to regenerate itself [26]. It is characterised by excessive deposition of extracellular matrix (ECM) proteins in response to chronic liver injury [27]. In addition to steatotic diseases such as MASLD or MASH, chronic liver injury can also be caused by viral infections (e.g., HBV and HCV), prolonged exposure to hepatotoxic substances (e.g., ethanol, acetaminophen) or bacterial toxins (mainly lipopolysaccharide, LPS) [28,29,30]. The development of fibrosis is a complex and multifaceted process involving intensive interactions between hepatocytes, hepatic stellate cells (HSCs) and immune cells (e.g., Kupffer cells). Many signalling pathways, including reactive oxygen species (ROS), platelet-derived growth factor (PDGF) and transforming growth factor-β (TGF-β), are involved in the activation of HSCs and their transformation into myofibroblasts (MFBs), which in turn express high levels of ECM proteins and alpha-smooth muscle actin (α-SMA) [31]. Excessive synthesis of ECM proteins, such as type I and III collagen, results in the development of fibrous scarring, which can ultimately disrupt proper liver function [32]. Many therapeutic approaches have been proposed, including synthetic small molecules (e.g., pegbelfermin), dietary supplementation, immunotherapeutics (e.g., simtuzumab) and even genetic regulation (e.g., non-coding *RNAs*), but none of them show sufficient efficacy to alleviate liver fibrosis [33]. On the other hand, it is well documented that plant secondary metabolites, including flavonoids, are widely used as therapeutic agents due to their high efficacy and relatively mild side effects. The presence of significant quantities of these substances has been well documented in a wide variety of common edible plants and medicinal herbs. In addition, a considerable amount of research has been carried out on the chemistry of flavonoids. In particular, the antioxidant and anti-inflammatory properties of flavonoids appear to be most relevant for their potential use as antifibrotic and hepatoprotective agents [9,34].

## 3. Sources and Biological Functions of Kaempferol

**Figure 1:**
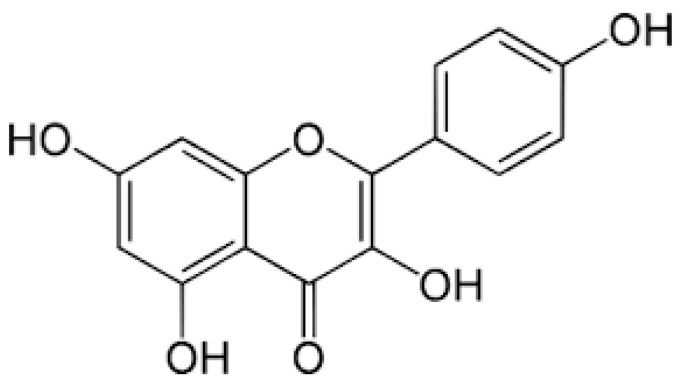
Chemical structure of kaempferol.

Kaempferol (3,5,7-trihydroxy-2-(4-hydroxyphenyl)-4H-chromen-4-one, molecular formula C_15_H_10_O_6_, molecular weight 286.24 g/mol) is a secondary plant metabolite that belongs to the larger group of flavonoids. They are polyphenolic molecules that share a basic structure of phenyl-benzo-γ-pyran, where aromatic ring A is condensed with heterocyclic ring C and linked to another aromatic ring B [35]. This group of compounds has strong antioxidant properties, mainly due to the phenolic hydroxyl groups attached to the aromatic rings [36]. In the realm of flavonoids, kaempferol, a member of the flavanol sub-group, has emerged as a subject of considerable scientific interest in recent times. This compound was first discovered in the tea plant (*Camellia sinensis*), but its name comes from Engelbert Kaempfer, a 17th-century German naturalist and physician who contributed significantly to the introduction of Japanese botanical knowledge in Europe [37]. Kaempferol has a polyphenolic structure with a carbonyl group at position 4 and four hydroxyl groups at positions 3, 4′, 5, and 7 (another name: 3,4′,5,7-tetrahydroxyflavone). The pure substance is a yellow crystalline powder with moderate solubility in water, but soluble in hot ethanol, ether, and hydroxide solutions [38]. The chemical structure of kaempferol is shown in Figure 1.

Kaempferol is widely distributed in various dietary and medicinal plants. It is one of the major flavanols that contribute to the beneficial effects of a plant-based diet [39]. Several vegetables, fruits and traditional medicinal herbs are particularly rich in kaempferol. The most significant amounts of kaempferol can be found in vegetables such as kale, spinach, onions, or beverages, especially black or green tea infusions [39]. Selected major sources of kaempferol are listed in Table 1.

Flavanols, such as kaempferol, have long been recognised as compounds with potent antioxidant properties [44], but a number of studies indicate that polyphenols (including kaempferol), rather than acting solely as direct antioxidants, appear to exert their beneficial effects primarily by modulating cellular signalling pathways and molecular mechanisms that regulate cell function in both healthy and pathological conditions [45]. For this reason, polyphenols have been extensively studied as potential therapeutic agents in various pathologies, including neurodegenerative, metabolic or inflammatory diseases, as well as in oncology as anticancer agents [38,41,46,47]. In addition, recent studies show promising results for the use of kaempferol as a preventive and antidotal agent against various natural and man-made toxins [48]. Kaempferol has also been investigated as an antiviral, antimicrobial, antiprotozoal and antifungal compound [48,49]. In this review, we will focus on the use of kaempferol in various metabolic disorders, especially those related to liver function.

## 4. Metabolism and Bioavailability of Kaempferol

**Figure 2:**
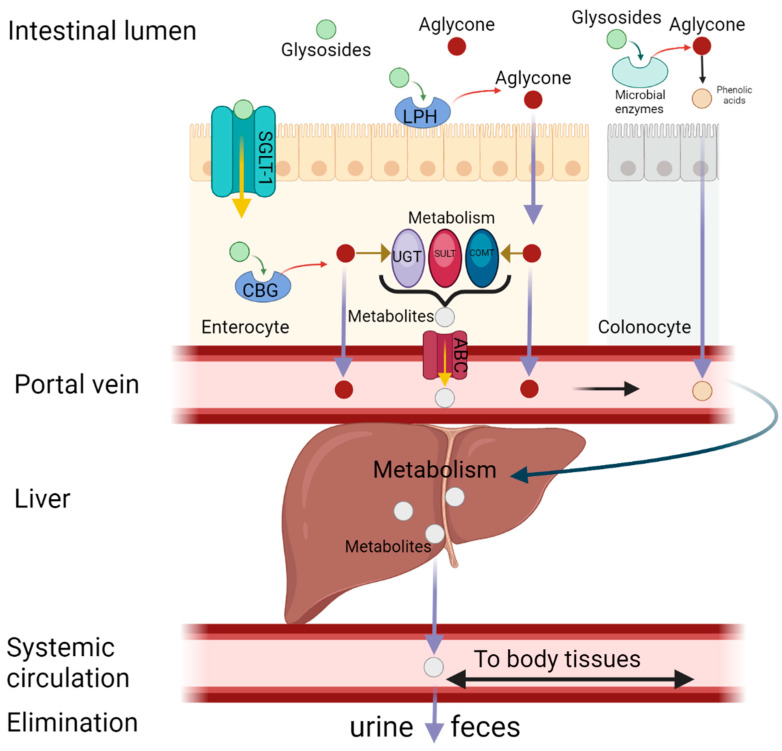
An overview of kaempferol ADME processes in the body. After ingestion, kaempferol may be directly transported to the enterocyte in the form of aglycone or glucosides as well as prior to absorption sugar moiety can be cleaved. Phase I and phase II metabolism occur either in enterocytes or in hepatocytes, and in the form of methyl, sulfur or glucuronide metabolites, kaempferol is distributed to the target tissues and ultimately excreted mainly in the renal way. Abbreviations: ABC—ATP-binding cassette, CBG—cytosolic β-glucosidase, COMT—catechol-O-methyltransferase, LPH—lactase-phlorizin hydrolase, SGLT-1—sodium-dependent glucose transporter-1, SULT—sulfotransferase, UGT—uridine-5ʹ-diphosphate-glucuronosyltransferase. Created in BioRender.

Flavonoids in general can occur in three chemical compositions: as aglycones, glycosides or methylated derivatives. Aglycone is a basic structure of any flavonoid, consisting of two benzene rings linked to a heterocyclic pyrene ring (the actual structure of kaempferol is shown in Figure 1). Glycosides consist of an aglycone and a sugar moiety, usually attached at the 3 or 7 position by a glycosidic linkage [50]. The most notable sugars that form glycosides with kaempferol include D-glucose, glucorhamnose, galactose, L-rhamnose, rutinose and arabinose [34]. There are examples of widely distributed kaempferol glycosides such as kaempferol-3-O-glucoside (astragalin) and kaempferol-3-O-rutinoside, found for example in tea and mulberry [51,52], or kaempferol-3-O-β-d-glucopyranoside-6-p-coumaril ester (tiliroside), found in lime or rose [53]. However, many other glycosides are found only in certain plant families or species and are therefore characteristic of these taxa and may be responsible for their unique properties [54]. Kaempferol in aglycon form has different physicochemical properties (e.g., lipophilicity, pKa, molecular size, solubility, etc.) than its glycosides, resulting in different pharmacokinetic properties, so the bioavailability of certain chemical forms of kaempferol may vary. Kaempferol in the aglycone form has lipophilic properties, whereas its glycosides become lipophobic when combined with sugars [55]. Nevertheless, either the hydrophobic aglycone or the hydrophilic glycosides of kaempferol are mainly supplied to the human body by the oral route, as they are very common phytochemicals present in many dietary products [39]. However, the bioavailability of the various chemical forms of oral kaempferol is low and has been calculated to be around 2% [46]. In the aglycon form, kaempferol is absorbed unchanged by passive diffusion into the enterocyte. Highly polar glycosides can be hydrolysed by the enzyme lactase-phlorizin hydrolase (LPH), which is present at the intestinal brush border. As a result, aglycon is released from its glycoside form and passively diffuses into the intestinal cells. The colonic microbiota, such as *Escherichia coli*, may also play an important role in the hydrolysis of kaempferol glycosides, but the cleaved aglycones in turn undergo further biotransformation to phenolic acids and are absorbed in this form [56,57]. However, glycosides can also be absorbed directly into the enterocyte via active transport mediated by sodium-dependent glucose transporter-1 (SGLT-1) and subsequently hydrolysed by the cytosolic enzyme β-glucosidase (CBG) [58]. Aglycones present in enterocytes may be transported directly into the portal circulation or may be metabolised prior to further distribution. Biotransformation in enterocytes involves both phase I (oxidation and O-demethylation) and phase II (sulphatation, glucuronidation, and methylation) reactions [59]. The main enzymes involved in phase II enzymes are uridine-5ʹ-diphosphate-glucuronosyltransferases (UGT), sulfotransferases (SULT) and catechol-O-methyltransferases (COMT) [56]. Unmetabolized aglycons and kaempferol metabolites are transported into the hepatic portal vein via passive diffusion and ATP-binding cassette (ABC), respectively [58]. The remaining kaempferol aglycones are metabolised in the liver during phase I and phase II metabolism and distributed to target organs, tissues and cells via the systemic circulation in the form of methyl, sulphur or glucuronide (mainly 7-O-glucuronide) [60]. These polar metabolites are mainly excreted by the kidneys in the urine or by the bile in the faeces. Approximately 2–2.5% of the total kaempferol ingested is excreted unchanged in the urine [61]. Extensive first-pass metabolism is probably responsible for the low bioavailability of kaempferol (approximately 2%), which limits its clinical use [46]. However, there are some modern approaches (nanoparticles, structural modifications, chimeric molecules) that could certainly be exploited to improve kaempferol bioavailability [62,63]. A schematic representation of the absorption, metabolism, distribution and excretion (ADME) of kaempferol is shown in Figure 2.

## 5. Effect of Kaempferol on Liver Condition

A substantial body of research has demonstrated the hepatoprotective properties of kaempferol. However, it is important to note that the preponderance of extant evidence derives from preclinical models [64,65,66]. Pretreatment with kaempferol in rats subjected to carbon tetrachloride administration has been shown to enhance liver enzyme activity and mitigate liver damage in rats treated with acetaminophen, an effect that is associated with an augmentation in sirtuin 1 (SIRT1) activity [66]. Recent research has highlighted the critical protective function of SIRT1 within the liver. The enzyme in question has been shown to influence a variety of physiological processes, including apoptosis, cel viability and antioxidant concentrations [67]. This NAD+-dependent deacetylase has been found to mediate these effects by deacetylation of transcription factors (e.g., NF-κB and STAT3) involved in the processes of inflammation and the maintenance of antioxidant potential, as well as fork-head transcription factors (FOXO and p53) and PGC-1 (which is involved in mitochondrial biogenesis). SIRT1 increases cell proliferation and survival by deacetylating multiple transcription factors. It also reduces oxidative stress and cellular inflammation as well as increases the production of ATP and mitochondrial biogenesis [68]. By increasing antioxidant levels and reducing inflammation and apoptosis in the liver, kaempferol has the capacity to provide comprehensive protection against oxidative damage to this organ [69].

**Figure 3:**
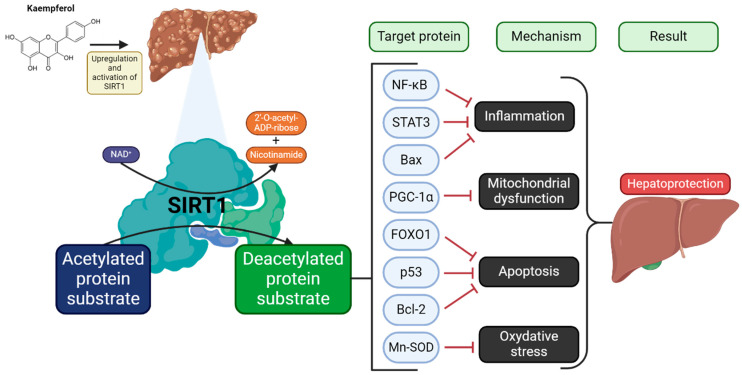
An overview of upregulation and activation of SIRT1 in the liver by kaempferol. For further explanations please see text above. Abbreviations: Bax—bcl-2-like protein 4, Bcl-2—B-cell CLL/lymphoma 2, FOXO1—Forkhead box protein O1, Mn-SOD—manganese-dependent superoxide dismutase, NF-κB—nuclear factor kappa-light-chain-enhancer of activated B cells, p53—tumour protein p53, PGC-1α—peroxisome proliferator-activated receptor gamma coactivator 1-alpha, SIRT1—sirtuin-1, STAT3—signal transducer and activator of transcription 3. Inhibition (⟞). Adopted and modified from figures by Yang et al. [68] and Alkandahri et al. [71]. Created in BioRender.

As postulated by BinMowyna and AlFaris [70], the hepatoprotective effect of the compound is a consequence of the deacetylation of FOXO1. The suppression of the expression of a number of apoptotic genes has been observed to be accompanied by an increase in the expression of antiapoptotic and antioxidant genes (MnSOD and Bcl-2). This has been shown to lead to acetylation and inactivation of NF-κB p65, inhibition of p53 acetylation, nuclear translocation and a consequent increase in Bax synthesis. NF-κB p65 generally stimulates inflammation by upregulation of inflammatory cytokines and induces apoptosis through Bcl-2 suppression and Bax upregulation. Upregulation and activation of SIRT1 in the liver by kaempferol are shown in Figure 3 [68,71].

The hepatoprotective effects of kaempferol are attributed to its specific modulation of liver-related pathways, including SIRT1 activation, CYP2E1 inhibition, TLR4/NF-κB suppression and ALK5/Smad pathway interference. Unique mechanisms distinguish it from other flavonoids such as quercetin and genistein, and highlight its potential as a targeted therapeutic agent for liver disease. Quercetin shares some antioxidant and anti-inflammatory properties, but does not have the same level of specificity in modulating SIRT1 activity or inhibiting CYP2E1 [72]. Genistein, primarily known for its estrogenic effects, lacks the direct involvement in liver-specific pathways that kaempferol demonstrates [73]. These specific pathways highlight kaempferol’s potential as a therapeutic agent for liver diseases, and warrant further clinical investigation to fully elucidate its efficacy and safety.

### 5.1. Reduction of Hepatic Lipid Accumulation

Two mechanisms that regulate lipid levels are lipid storage and metabolism. An imbalance in either process can lead to the accumulation of lipids in the liver, potentially causing harm to health [74]. As demonstrated by in vitro studies conducted with oleic acid-induced HepG2 cells, treatment with kaempferol led to a considerable decrease in lipid storage [74]. In addition, results from an in vivo study of type 2 diabetes in mice substantiated that a dosage of kaempferol (50 mg/kg) led to a marked diminution of lipid accumulation and a significant amelioration of liver damage, which is a key area of concern. New research has shown that SIRT1 and AMPK (AMP-activated protein kinase) work together to form a positively regulated loop that reinforces each other [75]. Nevertheless, research has indicated that the progression of hepatic steatosis leads to the inhibition of SIRT1/AMPK [76]. The activation of SIRT1/AMPK signalling has been demonstrated to promote fatty acid oxidation (FAO) through the modulation of the proliferator-activated receptor gamma coactivator 1α (PGC1α), acetyl-CoA carboxylase (ACC) and carnitine palmitoyltransferase 1 (CPT1) pathways. In mice, decreased hepatic AMPK activity has been found to lower ACC phosphorylation, which inactivates ACC, thereby reducing fatty acid oxidation (FAO) and increasing lipogenesis [77]. Sterol regulatory element-binding proteins (SREBPs) are essential transcription factors that control the expression of genes responsible for lipid, cholesterol, fatty acid, and triglyceride production in liver tissues [78]. Research has shown that AMPK suppresses SREBP activation by promoting its deacetylation, which in turn lowers the expression of crucial lipogenic genes like fatty acid synthase (*FASN*) [79]. Kaempferol treatment caused activation of SIRT1 and AMPK, a significant improvement in the concentrations of fatty acid oxidation-activated PGC1α and a reduction in the concentrations of lipid synthesis-related proteins. In addition, the anti-lipid effect of kaempferol was blocked when SIRT1 or AMPK were silenced. This suggests that kaempferol could be a therapeutic agent for controlling lipid accumulation in the liver by targeting the SIRT1/AMPK signalling pathway.

### 5.2. Inhibition of Hepatic Inflammation

**Figure 4:**
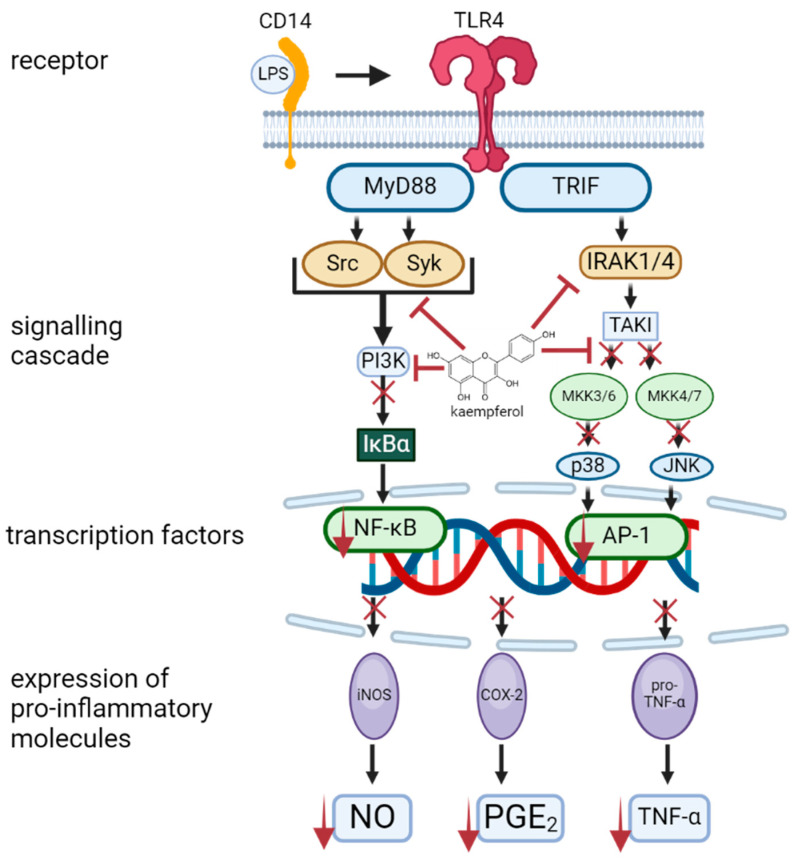
An overview of kaempferol protection mechanism in LPS-induced acute liver injury. For further explanations please see text above. Inhibition (⟞). Adopted and modified from figures in Alkandahri et al. [71]. Created in BioRender.

The liver is vulnerable to the toxic effects of chemicals, which can result in liver damage, fibrosis, and impaired function [71]. One such substance that has been shown to induce liver damage and trigger receptors such as TLR4 in hepatocytes and Kupffer cells is lipopolysaccharide (LPS) [80]. It has been demonstrated that this compound has the capacity to promote NF-κB phosphorylation and the production of pro-inflammatory cytokines such as TNF-α, IL-1 and IL-6 [81]. It has been demonstrated that LPS-induced hepatocellular injury can also lead to oxidative and nitrosative stress, resulting in the elevation of oxidants like ROS and RNS, depletion of endogenous antioxidants such as GSH and SOD, and elevation of MDA [82]. A multitude of cells activate a wide range of intracellular signalling cascades via two key adaptor molecules containing Toll/IL-1 receptor domains, TRIF and MyD88, to promote the activation of pro-inflammatory transcription factors including NF-κB and AP-1. This process results in the expression of genes that are involved in the inflammatory response, including iNOS, COX-2, cytokines, and chemokines [83]. It has been reported that kaempferol significantly decreases the expression level of *TLR4 mRNA* and protein to reduce NF-κB p65 phosphorylation in liver tissue. Kaempferol can also suppress the production and expression of COX-2, IL-1β, TNF-α, and *IL-6 mRNA*, which play key roles in inflammation. Additionally, it reduces the levels of NO and PGE2 while lowering *iNOS mRNA* expression in cases of acute liver injury. These findings indicate that kaempferol alleviates liver inflammation by inhibiting TLR4 and NF-κB activation, thereby reducing proinflammatory cytokine production. Moreover, it protects against hepatic nitrosative stress and helps restore normal liver function [84]. A mechanism of the protection of kaempferol against LPS-induced acute liver injury is illustrated in Figure 4 [71].

Persistent fat accumulation in the liver leads to excessive production of the extracellular matrix, surpassing its breakdown rate and ultimately causing progressive liver fibrosis [46]. Liu et al. [85] investigated this process using an in vitro model of oleic acid-treated HepG2 cells and an in vivo model of high-fat diet (HFD)-fed Sprague-Dawley (SD) rats. Their findings suggest that kaempferol (1 or 10 μM) can enhance NF-κB levels in the cytoplasm while reducing its presence in the nucleus, thereby lowering TNF-α and IL-6 levels- key factors in the pathophysiology of MASLD. Additionally, kaempferol (20, 40, or 60 μM) was shown to alleviate liver fat accumulation by modulating endoplasmic reticulum stress (ERS) and the expression of liver X receptor (LXR) and lysophosphatidylcholine acyltransferase 3 (LPACT3). These mechanisms are thought to play a crucial role in the development of hepatic steatosis and inflammation. Reducing liver inflammation may be achieved by suppressing the *mRNA* expression of inflammatory markers such as TNF-α and IL-6 [86].

### 5.3. Inhibition of Hepatic Oxidative Stress

It is imperative to note the intricate relationship between oxidative stress, inflammation and apoptosis. These processes are of particular significance within both physiological and pathological conditions [87]. Kaempferol has been shown to play a role in the treatment of diseases through its antioxidant potential. In a study on diabetic rats, it was observed that administration of kaempferol led to a significant improvement in glucose, insulin, and lipid peroxidation product levels in the plasma [88]. Furthermore, the levels of enzymatic and non-enzymatic antioxidative substances were found to be restored to nearly normal levels [89].

In a further study [90] the activity of SOD and the levels of MDA were determined in the liver to investigate the role of kaempferol in the oxidative response following haemorrhagic shock. The results obtained demonstrated that, in comparison to the sham group, the haemorrhagic shock groups exhibited significantly elevated levels of MDA. Conversely, SOD activity was reduced in the haemorrhagic shock groups in comparison to the control group. The injection of kaempferol after haemorrhagic shock had no effect on liver MDA levels and SOD activity when compared with the haemorrhagic shock group. However, when kaempferol was injected 12 h prior to the induction of haemorrhagic shock, increased SOD activity and decreased MDA levels in the liver were observed when compared with the haemorrhagic shock group. Furthermore, a significant decrease in plasma levels of IL-6 and TNF-α was observed, accompanied by the restoration of MDA, SOD, and MPO levels in the liver. Concurrently, an augmented expression of HO-1 was observed. Collectively, these observations suggest that kaempferol may possess the potential to mitigate the detrimental consequences of haemorrhagic shock in murine models. It is also notable that oxidative stress can be induced by excessive alcohol consumption. Ethanol is metabolised by alcohol dehydrogenase (ADH) and CYP2E1 to produce ROS. A recent study demonstrated a correlation between CYP2E1 activity and ethanol-induced liver damage and lipid peroxidation [91].

Elevated levels of liver enzymes (AST and ALT) have been shown to damage liver cells. These enzymes rely on pyridoxal phosphate (PLP) to function and play a key role in converting aspartate and ketoglutarate into glutamate and oxaloacetate. When liver cells are damaged, the levels of these enzymes can rise, indicating a loss of membrane integrity and impaired cellular function. Furthermore, it has been demonstrated that oxidative stress can induce Nrf2 in human hepatocyte cells by facilitating dissociation from Keap1 and subsequent translocation to the nucleus, where it binds to antioxidant response elements and activates target gene expression [92].

It has been demonstrated that kaempferol has the capacity to inhibit CYP2E1 at both the expression and activity levels, consequently leading to a reduction in ROS levels and liver damage. The significant decrease in serum AST and ALT levels is due to this inhibitory effect. The induction of reactive antioxidant enzymes (GSH and SOD) by this compound serves to remove lipid products (MDA) and ROS (H_2_O_2_) [93]. Furthermore, kaempferol has been shown to induce protective effects on liver structure by inhibiting hepatocyte apoptosis through the reduction of apoptosis-related proteins, including cytochrome c, Bax, Bcl-2, caspases:3, 8 and 9 [94].

### 5.4. Down-Regulation of Liver Fibrosis

The process of liver fibrosis is characterised by persistent or recurrent liver damage resulting from hepatotoxic substances such as alcohol, in addition to chronic liver diseases including alcoholic hepatitis, steatosis, and viral hepatitis, along with autoimmune disorders [95]. Cirrhosis, frequently associated with liver failure, represents the culmination of chronic liver necrosis and serves as the ultimate outcome of the aforementioned process [96]. Liver fibrogenesis is primarily driven by dysfunctional hepatic stellate cells (HSCs), which, under normal physiological conditions, act as stores of vitamin A in their inactive form. However, upon activation, these cells transform into myofibroblast-like cells, express α-SMA and produce large amounts of collagen. Accumulation of collagen results in the replacement of normal liver parenchyma by scar tissue, leading to liver fibrosis [97]. TGF-β is a regulatory cytokine that plays a critical role in the process of liver fibrosis, affecting HSC activation and proliferation, and ECM formation [98]. As demonstrated in the relevant literature, transforming growth factor-beta (TGF-β) has been shown to bind to its cognate receptor, namely TGF-β type II, thus resulting in the phosphorylation of Smad2 and Smad3. This process, in turn, has been observed to activate hepatic stellate cells (HSCs) and initiate the transcription of pro-fibrosis genes [99].

Kaempferol has been shown to be capable of inhibiting type I collagen expression in HSCs and reducing collagen density in liver tissue. It has been demonstrated that this can be achieved by reducing the phosphorylation of Smad2 and Smad3 by the serine/threonine kinase, attenuating α-SMA production, and inhibiting TGF-β-stimulated HSCs. In addition, it has been shown to bind specifically to ALK5 and further inhibit the TGF-β/Smad pathway. It may also act as an anti-fibrotic agent against fibrotic diseases [100].

### 5.5. Modulation of Gut Microbiota

Hepatic steatosis occurs when excess fat accumulates in the liver. This condition is frequently seen in individuals with obesity and type 2 diabetes. Extensive research involving both human and animal studies has shown a strong link between obesity and the gut microbiome [101,102].

Recent findings suggest that polyphenols, such as kaempferol, can influence gut microbiota composition, potentially improving metabolic disorders [103]. In the study conducted by Wang et al. [104] C57BL/6 J mice were fed a high-fat diet and given kaempferol (200 mg/kg) for eight weeks. The results showed a reduction in body weight and fat deposits in various white adipose tissues, including inguinal, epididymal, and perirenal fat. Additionally, kaempferol supplementation increased the Shannon index in faecal samples, indicating greater microbial diversity. The treatment also altered the gut microbiome by increasing the relative abundance of Bacteroidetes and Proteobacteria while reducing Firmicutes at the phylum level. At the genus level, there was an increase in Akkermansia, Bacteroides, and Lactobacillus populations. Another study extended the supplementation period to 16 weeks in obese C57BL/6 J mice and found that kaempferol helped counteract obesity-related changes in gut microbiota. The researchers observed an overall increase in microbial diversity in mice consuming a high-fat diet with kaempferol supplementation [105]. Results of kaempferol administration on on liver diseases in preclinical models and its mechanisms of action are presented in Table 2.

## 6. Safety of Kaempferol and Its Possible Interactions with Conventional Medicines

It should be noted that kaempferol exhibits a range of biological activities, some of which, depending on the circumstances, may be beneficial or detrimental. Kaempferol has been reported to have mutagenic and genotoxic properties in experiments on *Drosophila melanogaster* [121]. In vitro, kaempferol may induce chromosomal aberrations in V79 Chinese hamster cells, as I. Duarte Silva et al. have found [122]. This is thought to involve the biotransformation of kaempferol to quercetin by cytochromes P450 in the presence of metabolic activation systems. Moreover, it has been reported that kaempferol may result in a multitude of adverse consequences. For instance, as demonstrated by Lemos et al. [123], it was found that kaempferol could moderately inhibit the uptake of folic acid in human colon adenocarcinoma Caco-2 cells. This may have certain adverse impacts on folate-deficient individuals. It has been demonstrated that kaempferol exerts potent cytotoxic and anti-proliferative activities against several human cancer cells. A growing body of research has recently demonstrated that kaempferol exhibits selective toxicity towards cancer cells, while sparing normal cells. This finding serves to reinforce the notion that further investigation into kaempferol as an anticancer agent is warranted. For instance, the toxicity of kaempferol was significantly higher in HeLa cells than in normal HFF cells. This is manifested by the IC50 values of kaempferol HeLa cells being 45.63, 22.87 and 10.48 mM at 24, 48, and 72 h of treatment, respectively, as compared with 1079.0 and 707.0 mM for HFF cells at 48 and 72 h, respectively. At 24, 48 and 72 h, the IC50 values for HeLa cells were 45.63, 22.87 and 10.48 mM, compared with 1079.0 and 707.0 mM for HFF cells at 48 and 72 h. Mechanically, kaempferol induced cellular apoptosis and ageing by downregulating the PI3K/AKT and hTERT pathways [123]. The present result is consistent with the findings of Tu et al. [124], who demonstrated that kaempferol inhibited the proliferation of SiHa cervical cancer cells in a dose- and time-dependent manner. However, the study also revealed that kaempferol had minimal cytotoxic effects on normal kidney HK-2 cells. Examples of IC 50 for various cells are as follows: HepG2—30.0 μM (48 h), pancreatic cancer Mia PaCa-2—79.07 μM (48 h), lung cancer H460—50.0 μM (48 h), blood cancer HL60 cells- 250.60 μM (48 h), noncancer cells HFF—1079.00 μM (48 h). The findings of the studies indicated that kaempferol does not demonstrate significant toxicity towards normal cells, provided that the administered dose and the duration of administration are within reasonable limits.

When considering the potential interactions of kaempferol with conventional drugs, there are several factors to be taken into account, including its metabolic pathways and the manner in which it may affect drug-metabolising enzymes, particularly those located in the liver. The liver plays a pivotal role in the process of drug metabolism through the action of enzymes, including the cytochrome P450 (CYP) system. Kaempferol interacts with cytochrome P450 enzymes, including CYP3A4, which is key to drug metabolism. It has been demonstrated that drugs metabolised by this enzyme, including statins (e.g., atorvastatin) and certain immunosuppressants (e.g., cyclosporine), may experience increased plasma concentrations when co-administered with kaempferol, potentially resulting in adverse effects [125]. It may also inhibit CYP1A2, an enzyme that facilitates the metabolism of drugs such as theophylline and caffeine. There is some evidence to suggest that kaempferol can inhibit CYP2C9, which is involved in the metabolism of drugs like warfarin. This could increase the risk of bleeding if these medications are used concomitantly [126]. These interactions have the potential to result in alterations to drug levels in the bloodstream, either increasing toxicity or reducing the therapeutic efficacy of certain medications.

P-glycoprotein (P-gp) is a drug transporter that plays a role in limiting the absorption and enhancing the elimination of various drugs. It has been demonstrated that kaempferol is capable of inhibiting P-gp, which may consequently result in an enhancement of the bioavailability of drugs that are P-gp substrates. It has been hypothesised that this may result in an increased risk of adverse effects or toxicity in medications such as tacrolimus (utilised in the context of organ transplantation) or anticancer agents, including paclitaxel [127]. The influence of kaempferol on the expression of liver enzymes, particularly through the activation of nuclear receptors such as Pregnane X Receptor (PXR) and Aryl Hydrocarbon Receptor (AhR), suggests the potential for regulation of drug-metabolising enzymes [128,129]. This modulation has the potential to induce alterations in the hepatic metabolism of various pharmaceutical agents, which may necessitate dose recalibrations for patients receiving drugs that are substrates of these pathways [130].

## 7. Conclusions and Perspectives

Natural medicines are gaining worldwide recognition due to their excellent therapeutic efficacy and relatively low side effects. Kaempferol, a widely available natural compound, is present in many medicinal plants. This review provides an overview of its pharmacological effects on liver diseases, the molecular mechanisms involved, its dietary sources, and its pharmacokinetic properties. Various in vivo and in vitro studies have demonstrated that kaempferol exerts therapeutic effects on liver diseases by regulating metabolic functions and pathological processes. For instance, it can help reduce fat accumulation in the liver, decrease liver fibrosis, and support a healthy balance of intestinal flora, which plays a crucial role in its beneficial effects. In addition, the anti-inflammatory effect through the downregulation of pro-inflammatory cytokines is the common mechanism of action of kaempferol in the treatment of liver problems, including IL-6, IL-1β and TNF-α. Through multiple mechanisms and signalling pathways, these studies show that kaempferol has the potential to improve the symptoms of liver disease. It is evident that kaempferol is a natural compound that merits rigorous investigation. The present study, however, is not without its limitations. The research conducted on kaempferol in the context of liver disease researchers has been primarily confined to fundamental investigations, encompassing animal and cell experiments. Hence, the necessity for additional clinical studies arises, with the objective of substantiating the purported beneficial effects of kaempferol.

Natural compound safety assumes equal significance to its efficacy in the development of therapeutic drugs. While the majority of studies have shown that it is relatively non-toxic to normal cells under reasonable doses and times, the need for additional, longer-term clinical trials to better determine its safety in humans is crucial. Furthermore, there remains a paucity of data on the oral bioavailability of kaempferol, a key obstacle to its use in treating metabolic diseases. Enhancing the oral bioavailability of kaempferol through nanotechnology or structural modifications could help advance it from basic research to clinical use.

In summary, kaempferol holds significant promise as a treatment for liver diseases. However, further research, particularly clinical studies, is needed to provide stronger evidence for its proposed molecular mechanisms and targets. Additionally, well-structured, extensive, and long-term clinical trials are essential to assess its effectiveness and safety, ultimately supporting its transition into clinical practice and allowing more patients to benefit from its therapeutic potential.

## Figures and Tables

**Table 1:** Major dietary sources of kaempferol.

	Plant	Scientific Name	Amount	References
	Green chili	*Capsicum annum*	39	[40]
Plant extracts [mg/kg dry mass]	Onion leaves	*Allium fistulosum*	832	
	Papaya shoots	*Carica papaya*	453	
	Brinjal	*Solanum melongena*	80	
	Pumpkin	*Cucurbita maxima*	371	
	Sengkuang	*Pachyrrhizus erosus*	37	
	Carrot	*Daucus carota*	140	
	White radish	*Raphanus sativus*	38	
	Daun turi	*Sesbania grandifolia*	21	
	Lemon grass	*Cymbopogon citratus*	178	
	Cekur manis	*Sauropus androgynus*	323	
	Pegaga	*Hydrocotyle asiatica*	20	
	Bunga kantan	*Phaeomeria speciosa*	286	
	Black tea	*Camellia sinensis*	118	
	Beans	*Phaseolus vulgaris*	14	[41]
	Broccoli	*Brassica oleracea var. italica*	72	
	Cauliflower	*Brassica oleracea var. botrytis*	270	
Plant-derived beverages [μg/mL]	Lemon juice	*Citrus limon*	1.9	[42]
	Grapefruit juice	*Citrus × paradisi*	1.1	
	Pineapple juice	*Ananas comosus*	1.2	
	Apple juice	*Malus domestica*	1.0	
	Black tea	*Camellia sinensis*	11.4	[43]
Plants [mg/100 g fresh weight]	Spinach	*Spinacia oleracea*	7.86	[43]
	Garden cress	*Lepidium sativum*	13.00	
	Broccoli	*Brassica oleracea var. italica*	5.65	
	Kale	*Brassica oleracea var. sabellica*	5.65	
	Onion	*Allium cepa*	26.74	
	Rabbiteye blueberries	*Vaccinium virgatum*	2.36	

**Table 2:** Role of kaempferol in regulation of liver diseases.

Diseases	Model	Doses	Mechanism of Action/Effect	Ref.
MASLD	Male ddY mice	4.9 mg/kg	↓ TBARS and TNF-α caused by CCl_4_ free radicals	[66]
	C57BLKS/J mice fed HFD	87.5 µmol/kg	Regulation of hepatic lipid accumulation (activation of the SIRT1/AMPK pathway)	[74]
	HFD-induced SD rats	350 µmol/kg	Prevention of advancement of simple fatty liver disease to non-alcoholic steatohepatitis (blocking the NF-κB pathway)	[85]
	HepG2 cells	1 or 10 µM		
	C57BL/6 J mice fed HFD	0.5 mL/100 g	Regulation of BA metabolism in the serum and liver (enhancing CYP27A1 and NTCP expression)	[106]
MASLD	HepG2 cells	50 mg/kg	↓ fat buildup in the liver, ↑ NF-κB signalling pathway, ↑ mitochondrial beta-oxidation, ↑ expression of CPT1A	[107]
Liver injury	Bosentan-induced rat liver injury model and HEK-293 cells	25 mg/kg and 1–150 µM	↓ OATP1B1 transporter (keeping AST and ALT levels stable)	[108]
	Male swiss albino rats	25 mg/kg	↓ lipid peroxidation caused by CCl_4_ free radicals	[109]
	Mice and HepG2 cells	250 and 500 mg/kg and 100, 200, and 400 µM	↓ AA + Fe-induced ROS, ↓ glutathione depletion	[110]
	ALI mice model	10 and 20 mg/kg	↓ antioxidant defence activity, ↑ lipid peroxidation and oxidative stress	[111]
Liver fibrosis	L02, LX2, and rats	20 µM	↓ caspase-3 protein levels, ↑ p-ERK1/2, PI3K and Bcl-xL protein expression in L02 cells; ↑proliferation of LX2 cells, ↑ Bax and cleaved caspase-8.	[112]
	HSCs/CCl_4_-induced mouse model	2–10 µmol/L	↓ hyaluronan, ALT, AST and Smad2/3, ↓ collagen synthesis and HSC activation; ↑activin receptor-like kinase 5	[100]
Liver cancer	HepG2 cells	10, 20, 40, and 80 µM	↑ ROS production, ↑ cytochrome c ↑ *PIG3 mRNA* and protein, ↓ mitochondrial membrane potential, ↓ f Bax/Bcl-2 and caspase-9 and -3	[113]
Hepatotoxicity	Male C57BL/6 mice	30 and 60 mg/kg	↓ ALT and AST, ↓ liver cell damage, ↑ antioxidant enzymes and apoptosis; ↓ NLRP3 and pro-inflammatory molecules	[114]
Obesity	Wild-type zebrafish	7.5, 15, and 30 µM	↑ adipogenesis	[115]
	The 3T3-L1 preadipocytes	60 µM	↑ lipolysis, ↓ adipogenesis	[116]
	Human mesenchymal fat cells	1, 10 or 25 µM	↑ lipolysis, ↓ adipogenesis	[117]
	The 3T3-L1 preadipocytes	2.5, 5, 10, 20 and 40 µM	↓ adipogenic transcription factors, ↑ PPARα-mediated signalling of FAO	[118]
	C57BL/6 J male mice fed HFD	43.75, 87.5, and 175 µmol/kg	Regulation of adipocyte thermogenesis via the *CDK6/RUNX1/UCP1* pathway	[119]
	C57BL/6 J male mice fed HFD	a high-fat diet with 0.1% kaempferol	↑ intestinal barrier integrity, ↓ intestinal inflammation by inhibition of TLR4/NF-κB pathway	[105]
	C57BL/6 mice fed HFD	350 µmol/kg	↑ gut microbiota and ↓ the progression of insulin resistance.	[104]
	C57BL/6 mice fed HFD	0.875 µmol/kg	Regulation of physiological processes concerning energy balance and inflammation	[120]

↑ upregulating/improving, ↓ downregulating/decreasing

## Data Availability

Not applicable.

## Abbreviations

The following abbreviations are used in this manuscript:

ABC	ATP-binding cassette
ACC	acetyl-CoA carboxylase
ADH	alcohol dehydrogenase
ADME	absorption, distribution, metabolism, excretion
AhR	Aryl Hydrocarbon Receptor
ALK5	activin receptor-like kinase 5
ALT	alanine transferase
AMPK	SIRT1/AMP-activated protein kinase
AST	aspartate aminotransferase
Bax	bcl-2-like protein 4
Bcl-2	B-cell CLL/lymphoma 2
CBGCGA	cytosolic enzyme β-glucosidasechlorogenic acid
COMT	catechol-O-methyltransferase
COX	cyclooxygenase
CPT1	carnitine palmitoyltransferase 1
CYP2E1	cytochrome P450 2E1
ECM	extracellular matrix
ERS	endoplasmic reticulum stress
FAO	fatty acid oxidation
FASN	fatty acid synthase
FOXO	forkhead box protein
GCG	glucagon
GIP	gastric inhibitory polypeptide
GLP-1	glucagon-like peptide 1
GSH	glutathione
HCC	hepatocellular carcinoma
HBV	hepatitis B virus
HCV	hepatitis C virus
HFD	high-fat diet
HO-1	heme oxygenase 1
HSC	hematopoietic stem cells
IL	interleukin
iNOS	inducible nitric oxide synthase
LPACT3	lysophosphatidylcholine acyltransferase 3
LPH	lactase-phlorizin hydrolase
LPS	lipopolysaccharide
LXR	liver X receptor
MASLD	metabolic dysfunction-associated steatotic liver disease
MDA	malondialdehyde
MFBs	myofibroblasts
MnSOD	manganese-dependent superoxide dismutase
MPO	myeloperoxidase
NAD+	oxidized nicotinamide adenine dinucleotide
NAFLD	non-alcoholic fatty liver disease
NF-κB	nuclear factor kappa-light-chain-enhancer of activated B cells
NO	nitric oxide
P-gp	P-glycoprotein
p53	tumour protein p53
PDGF	platelet-derived growth factor
PGC-1	peroxisome proliferator-activated receptor gamma coactivator 1
PGE	Prostaglandin E
PLP	pyridoxal phosphate
PXR	Pregnane X Receptor
RNS	reactive nitrogen species
ROS	reactive oxygen species
SGLT-1	sodium-dependent glucose transporter-1
SIRT1	sirtuin 1
SOD	superoxide dismutase
SREBPs	sterol regulatory element-binding proteins
STAT3	signal transducer and activator of transcription 3.
SULT	sulfotransferase
TLR4	toll-like receptor 4
TNF-α	tumour necrosis factor-alpha
UGT	uridine-5ʹ-diphosphate-glucuronosyltransferase
α-SMA	alpha-smooth muscle actin
